# Combined Analysis of the Chloroplast Genome and Transcriptome of the Antarctic Vascular Plant *Deschampsia antarctica* Desv

**DOI:** 10.1371/journal.pone.0092501

**Published:** 2014-03-19

**Authors:** Jungeun Lee, Yoonjee Kang, Seung Chul Shin, Hyun Park, Hyoungseok Lee

**Affiliations:** Division of Life Sciences, Korea Polar Research Institute, Incheon, South Korea; Niels Bohr Institute, Denmark

## Abstract

**Background:**

Antarctic hairgrass (*Deschampsia antarctica* Desv.) is the only natural grass species in the maritime Antarctic. It has been researched as an important ecological marker and as an extremophile plant for studies on stress tolerance. Despite its importance, little genomic information is available for *D. antarctica*. Here, we report the complete chloroplast genome, transcriptome profiles of the coding/noncoding genes, and the posttranscriptional processing by RNA editing in the chloroplast system.

**Results:**

The complete chloroplast genome of *D. antarctica* is 135,362 bp in length with a typical quadripartite structure, including the large (LSC: 79,881 bp) and small (SSC: 12,519 bp) single-copy regions, separated by a pair of identical inverted repeats (IR: 21,481 bp). It contains 114 unique genes, including 81 unique protein-coding genes, 29 tRNA genes, and 4 rRNA genes. Sequence divergence analysis with other plastomes from the BEP clade of the grass family suggests a sister relationship between *D. antarctica*, *Festuca arundinacea* and *Lolium perenne* of the Poeae tribe, based on the whole plastome. In addition, we conducted high-resolution mapping of the chloroplast-derived transcripts. Thus, we created an expression profile for 81 protein-coding genes and identified *ndhC*, *psbJ*, *rps19*, *psaJ*, and *psbA* as the most highly expressed chloroplast genes. Small RNA-seq analysis identified 27 small noncoding RNAs of chloroplast origin that were preferentially located near the 5′- or 3′-ends of genes. We also found >30 RNA-editing sites in the *D. antarctica* chloroplast genome, with a dominance of C-to-U conversions.

**Conclusions:**

We assembled and characterized the complete chloroplast genome sequence of *D. antarctica* and investigated the features of the plastid transcriptome. These data may contribute to a better understanding of the evolution of *D. antarctica* within the Poaceae family for use in molecular phylogenetic studies and may also help researchers understand the characteristics of the chloroplast transcriptome.

## Introduction

Chloroplasts are plant-specific organelles that conduct photosynthesis, providing essential energy for the synthesis of starch, fatty acids, pigments, and amino acids [1], [2]. Chloroplasts contain DNA and their own genetic information. In higher plants, chloroplast genomes exist as circular DNA, with the size ranging from 120 kb to 150 kb, and generally have a highly conserved quadripartite organization composed of two copies of inverted repeats (IRs), which separate the large single copy (LSC) and small single copy (SSC) regions [3], [4]. In vascular plants, chloroplast genomes usually contain 110–130 unique genes encoding 4 rRNAs, 30–31 tRNAs, and 80–90 proteins; these encode ribosomal proteins and RNA polymerase subunits involved in protein synthesis, thylakoid proteins, and the Rubisco large subunit for photosynthesis, as well as protein subunits for an NADH dehydrogenase complex, which mediates redox reactions [2], [5]. Advances in high-throughput sequencing technologies have resulted in the full sequences of organelle genomes from a growing number of organisms [6]. Currently, plastid genome resources with >420 records have been established. These provide a vast amount of high-resolution information that can be exploited in phylogenetic and ecological studies, making it possible to track the evolutionary history of a species after obtaining the full sequence of its chloroplast genome.

The grass family (Poaceae), which occurs in nearly every terrestrial habitat, is one of the most diverse angiosperm families, including approximately 10,000 species over 700 genera. To date, 38 chloroplast genomes of grass species [32 from the BEP (Bambusoideae, Ehrhartoideae, Pooideae) clade and 6 from the PACMAD (Panicoideae, Arundinoideae, Chloridoideae, Micrairoideae, Aristidoideae, and Danthonioideae) clade] have been deposited into the GenBank database, and recent studies have tried to reconstruct the phylogeny of the subfamilies and genera in the Poaceae family using whole sequences of chloroplast genomes [7], [8].

Extremophile plants have evolved tolerance overcoming unfavorable environmental conditions, such as freezing temperatures, drought, high salinity, and high UV radiance. The genetic information on such species provides clues for the evolutionary or geological history of the species, as well as resources for genetic engineering. Antarctic hairgrass (*Deschampsia antarctica* Desv.) is the only native grass species that thrives in the harsh environment of Antarctica [9]. As an extremophile, it may be useful as a source of genes associated with stress tolerance [10]. It has also been suggested as an ecological marker of global warming because of its successful adaptation to climate change and its rapid spread [10], [11]. Despite the importance of this terrestrially isolated plant, its phylogenetic position is still controversial [12]–[14], and available genetic resources are limited.

Here, we obtained the complete chloroplast genome sequence of *D. antarctica* by high-throughput sequencing and *de novo* assembly. By comparison with the chloroplast genomes from other representative members of the BEP clade, we explored the deep-phylogenetic relationship of *D. antarctica* to other grass species at the genomic level. In addition, using combinatorial analysis of the RNA-seq data, we conducted high-resolution mapping of the chloroplast-derived transcripts to a reference chloroplast genome to demonstrate transcriptome profiles of the coding and noncoding genes and the posttranscriptional processing by RNA editing in the chloroplasts of *D. antarctica*. These data may contribute to a better understanding of the evolution of *D. antarctica* within the Poaceae family and the characteristics of the chloroplast transcriptome.

## Methods

### Ethics Statement

This study including sample collection and experimental research conducted on these materials was according to the law on activities and environmental protection to Antarctic approved by the Minister of Foreign Affairs and Trade of the Republic of Korea.

### Plant Materials

*Deschampsia antarctica* Desv. (Poaceae) plants growing under natural conditions were collected in the vicinity of the Korean King Sejong Antarctic Station (62°14′29″S, 58°44′18″W) on the Barton Peninsula of King George Island and then transferred to the lab and grown hydroponically, supplemented with 0.5× Murashige and Skoog (MS) medium containing 2% sucrose under a 16∶8 h light:dark cycle with a light intensity of 150 μmol m^−2^ s^−1^ at 15°C, a temperature that results in high Rubisco activity in *D. antarctica* [15].

### DNA and RNA Sequencing

Total genomic DNA was extracted from leaf tissues using the DNeasy Plant Mini Kit (Qiagen, Valencia, CA, USA) according to the manufacturer's instructions. Total RNA was extracted from whole plants using the RNeasy Plant Mini Kit (Qiagen). For the small noncoding RNA library, total RNA was extracted from leaves using the mirVana Kit (Ambion, Austin, TX, USA). The quality of the RNA and DNA was checked on a Bioanalyzer 2100 (Agilent, Santa Clara, CA, USA). The libraries were prepared and sequenced according to the manufacturer's instructions (Illumina, San Diego, CA, USA). The DNA library was constructed using TruSeq DNA sample preparation kits and a single lane of an Illumina HiSeq2000 sequencer (PE, 2×101 bp). For the mRNA library, multiplex libraries were obtained using TruSeq RNA sample preparation kits, and the samples were sequenced in one lane of an Illumina HiSeq2000 sequencer (PE, 2×101 bp). The small RNA library was constructed using the TruSeq Small RNA Sample Prep Kit; the resulting single end library was sequenced in one lane of an Illumina GAIIX sequencer (SE, 1×35 bp). The files containing the sequences and quality scores of reads were deposited in the NCBI Short Read Archive, and the accession numbers are SRX465632 (genomic DNA-Seq), SRX465633 (mRNA-Seq), and SRX465634 (Small RNA-Seq).

### Genome Assembly, Annotation, and Sequence Analysis

After trim of low quality reads and adapters, the raw reads were aligned to 330 publicly available chloroplast genomes downloaded from NCBI organelle genome resources. *De novo* assembly was done with the collected chloroplast-related reads by Celera Assembler 6.1 (Celera Genomics, Alameda, USA). The assembled contigs were ordered with reference chloroplast genomes of two ryegrass species, *Lolium multiforum* (NC_019651) and *Festuca altissima* (JX871939), which were identified as the top-hit species when the input reads were blasted against the nr database. The gaps were filled by realignment of input reads using Geneious R6 v6.1.5 (Biomatters Ltd., Auckland, New Zealand) and PCR-based Sanger sequencing using primers designed for gap-flanking regions (Table S1). The sequences from the junction and highly variable region were validated by Sanger sequencing. The complete plastome was annotated using the online software DOGMA with default parameters [16]. Repeat sequences were analyzed using REPuter [17].

### Phylogenetic Analysis

Complete plastome sequences of nine Poaceae species (accession numbers are listed in Table S2) were aligned using the LAGAN program within the mVISTA online suite of computational tools [18]. Default parameters were applied, and the annotation framework of the perennial ryegrass chloroplast genome was used. The percentage identity between each plastome, all relative to that of *D. antarctica*, was subsequently visualized using an mVISTA plot [19]. The plastome-based phylogeny was reconstructed for the nine Poaceae species using the whole plastome alignment generated by LAGAN. The phylogenetic tree was constructed through the method of maximum parsimony, as implemented by MEGA 5.2 [20]. Sites with gaps or missing data were excluded from the analysis, and statistical support was achieved through bootstrapping using 1000 replicates.

### Transcriptome and Small Noncoding RNA Analysis

We analyzed in-house RNA-seq data libraries generated from two sets of RNAs (mRNA and small RNA), obtained as described above. For transcriptome analysis, we analyzed combined data sets of mRNAs and small RNAs. The reads of the combined data sets were mapped to the complete chloroplast genome, and the filtered reads were collected using the Bowtie 2.0 program with mismatch ≤2 bp [21]. The filtered reads were remapped according to the genome annotation using Cufflinks to calculate the fragments per kilobase of exon per million fragments mapped (FPKM) values of the transcripts and TopHat for alignment of transcript variants [22]. For small noncoding RNA analysis, we collected the reads in the size range of 20–24 nt from the small RNA data set. The size-filtered reads were mapped using Bowtie 2.0 with the criterion of zero mismatch. To search for RNA-editing sites in the chloroplast genome, putative target sites were predicted using two independent methods: 1) the PREP-chloroplast [23] search program using the chloroplast-genome sequence and 2) SAMtools/BCFtools, which calls single-nucleotide polymorphisms (SNPs) and indels by comparing transcripts against references [24]. After prediction, the candidate sites were manually examined in the transcriptome data using the Integrative Genomics Viewer (IGV) genome browser.

## Results

### Chloroplast Genome Assembly and Validation

Illumina paired-end sequencing produced 153,346,825 raw reads with a sequence length of 101 bp and a total base number of 15,488,029,325. After quality trim and alignment of the raw reads against the publicly available chloroplast genomes reported in NCBI, we collected 1,985,544 chloroplast-related paired reads with 191,735,269 bases. The subsequent *de novo* assembly resulted in 18 large contigs >3 kb (max: 50,269 bp, min: 3,046 bp). To order the contigs, the chloroplast genomes of *L. multiforum*, and *F. altissima* were used as references because these species were identified as the top-hit species when the input reads were blasted against the nr database. The resulting gaps were filled by alignment of the input reads using the Geneious program and PCR-based Sanger sequencing. The sequences from the junction regions (LSC–IRA, LSC–IRB, SSC–IRA, SSC–IRB) and the regions with high interspecific variability were validated by Sanger sequencing. The final *D. antarctica* chloroplast genome sequence has been submitted to GenBank (Accession No. KF887484).

### Genome Organization and Gene Content

**Figure 1:**
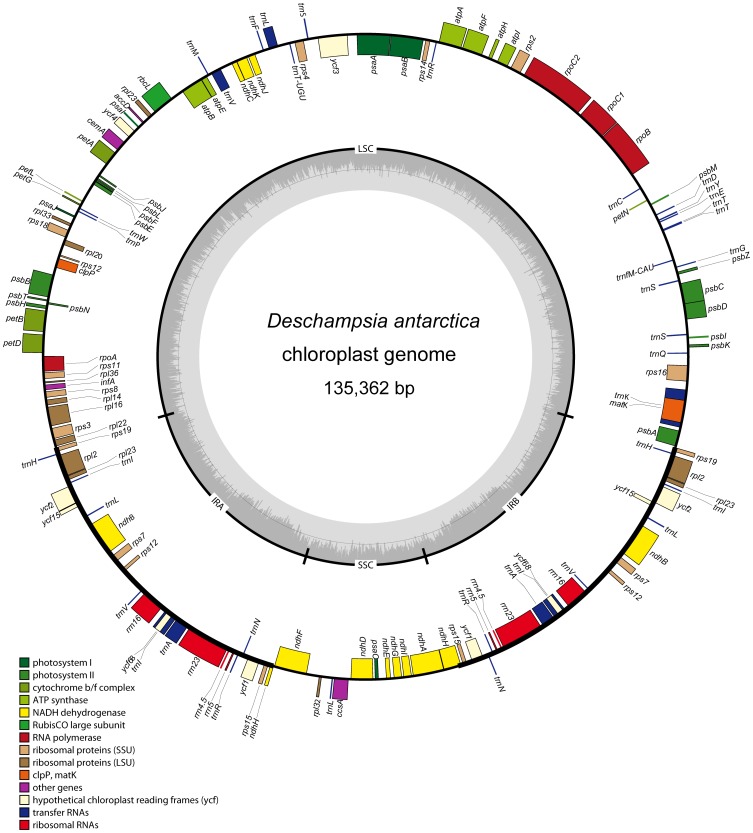
Map of the chloroplast genome of *Deschampsia antarctica*. Genes lying outside of the outer circle are transcribed clockwise, while those inside the circle are transcribed counterclockwise. Genes belonging to different functional groups are color coded. The innermost darker gray corresponds to GC, while the lighter gray corresponds to AT content. IR, inverted repeat; LSC, large single copy region; SSC, small single copy region.

The size of the *D. antarctica* chloroplast genome was 135,362 bp, similar in range as other Poaceae species, with a typical quadripartite structure (Figure 1). The LSC and SSC regions were 79,881 bp and 12,519 bp in size, respectively, separated by a pair of inverted repeats (IRa and IRb), which were both 21,481 bp in length. The GC content of the *D. antarctica* chloroplast genome was 38.3%, consistent with other reported Poaceae chloroplast genomes. The GC contents of the LSC and SSC regions were 36.3% and 32.4%, respectively, whereas that of the IR region was 43.85%.

The *D. antarctica* chloroplast genome contained 81 unique protein-coding genes, 12 of which were duplicated in the IR, including *rps7*, *rps12*, *rps15*, *rps19*, *rpl2*, *rpl23*, *ycf1*, *ycf2*, *ycf15*, *ycf68*, *ndhB*, and partial *ndhH*. Additionally, 29 unique tRNA genes, representing all 20 amino acids, were distributed throughout the genome (1 in the SSC region, 20 in the LSC region, and 8 in the IR region). Four rRNA genes were also identified, with complete duplication in the IR regions. Altogether, the *D. antarctica* chloroplast genome contained 114 unique genes (Table 1). Among them, 14 genes contained a single intron (9 protein-coding genes and 5 tRNA genes), while *ycf3* contained two introns. Of the 15 genes with introns, 10 were located in the LSC (7 protein-coding genes and 3 tRNAs; 9 contained one intron and 1 contained two introns), 1 in the SSC (a protein-coding gene with a single intron), and 4 in the IR region (2 protein coding genes and 2 tRNAs, all 4 containing a single intron) (Table 2). The *rps12* gene is a trans-spliced gene with a 5′-end exon located in the LSC region and duplicated 3′-end exons located in the IR region. The *trnK-UUU* gene contained the largest intron (2,486 bp), which included the *matK* gene.

**Table 1:** Genes present in the *Deschampsia antarctica* chloroplast genome.

Products		Genes
1	Photosystem I	*psaA*, *B*, *C*, *I*, *J*, *ycf3* a, *ycf4*
2	Photosystem II	*psbA*, *B*, *C*, *D*, *E*, *F*, *H*, *I*, *J*, *K*, *L*, *M*, *N*, *T*, *Z*
3	Cytochrome b6/f	*petA*, *B* b, *D* b, *G*, *L*, *N*
4	ATP synthase	*atpA*, *B*, *E*, *F* b, *H*, *I*
5	Rubisco	*rbcL*
6	NADH oxidoreductase	*ndhA* b, *B* b ^,^ c, *C*, *D*, *E*, *F*, *G*, *H* c, *I*, *J*, *K*
7	Large subunit ribosomal proteins	*rpl2* b ^,^ c, *14*, *16* b, *20*, *22*, *23* c, *32*, *33*, *36*
8	Small subunit ribosomal proteins	*rps2*, *3*, *4*, *7* c, *8*, *11*, *12* b ^,^ c ^,^ d, *14*, *15* c, *16* b, *18*, *19* c
9	RNAP	*rpoA*, *rpoB*, *C1*, *C2*
10	Other proteins	*accD*, *ccsA*, *cemA*, *clpP*, *matK*, *infA*
11	Proteins of unknown function	*ycf1* c ^,^ e, *ycf2* c, *ycf15* c, *ycf68* c
12	Ribosomal	*rrn23* c, *16* c, *5* c, *4.5* c
13	Transfer RNAs	*trnA(UGC)* b ^,^ c, *C(GCA)*, *D(GUC)*, *E(UUC)*, *F(GAA)*, *G(UCC)*, *H(GUG)* c, *I(CAU)* c, *I(GAU)* b ^,^ c, *K(UUU)* b, *L(UAA)* b, *L(UAG)*, *L(CAA)* c, *fM(CAU)*, *M(CAU)*, *N(GUU)* c, *P(UGG)*, *Q(UUG)*, *R(ACG)* c, *R(UCU)*, *S(GCU)*, *S(GGA)*, *S(UGA)*, *T(GGU)*, *T(UGU)*, *V(UAC)* b, *V(GAC)* c, *W(CCA)*, *Y(GUA)*

a Gene containing two introns.

b Gene containing a single intron.

c Two gene copies in the IRs.

d Gene divided into two independent transcription units.

e Pseudogene.

**Table 2:** Genes containing introns in the *Deschampsia antarctica* chloroplast genome and the length of the exons and introns.

Gene	Location	Length (bp)				
		Exon I	Intron I	Exon II	Intron II	Exon III
*rps16*	LSC	40	830	209		
*atpF*	LSC	159	802	408		
*ycf3*	LSC	126	749	228	728	159
*petB*	LSC	6	760	642		
*petD*	LSC	9	686	525		
*rpl16*	LSC	9	893	402		
*rps12* *	LSC	117	-	231		
*rpl2*	IR	393	660	432		
*ndhB*	IR	777	712	756		
*ndhA*	SSC	549	1012	540		
*trnK-UUU*	LSC	38	2486	33		
*trnL-UAA*	LSC	37	537	50		
*trnV-UAC*	LSC	39	605	37		
*trnI-GAU*	IR	42	801	35		
*trnA-UGC*	IR	38	811	35		

*rps12 is trans-spliced gene with 59 end exon located in the LSC region and the duplicated 39 end exon located in IR regions.

On the basis of the sequences of protein-coding genes and tRNA genes within the chloroplast genome, the frequency of codon usage was deduced (Table 3). Among these codons, 2,466 (11.22%) encode for leucine, while 321 (1.46%) encode for cysteine, which are the most and least used amino acids, respectively. The codon usage is biased toward a high representation of A and T at the third codon position, which is similar to a previous report [25].

**Table 3:** The codon–anticodon recognition pattern and codon usage in the *Deschampsia antarctica* chloroplast genome.

Amino acid	Codon	No.*	tRNA	Amino acid	Codon	No.	tRNA
**Phe**	UUU	790		**Tyr**	UAU	599	
**Phe**	UUC	448	trnF-GAA	**Tyr**	UAC	211	trnY-GUA
**Leu**	UUA	790	trnL-UAA	**Stop**	UAA	48	
**Leu**	UUG	445	trnL-CAA	**Stop**	UAG	20	
**Leu**	CUU	492		**His**	CAU	371	
**Leu**	CUC	226		**His**	CAC	164	trnH-GUG
**Leu**	CUA	363	trnL-UAG	**Gln**	CAA	572	trnQ-GUU
**Leu**	CUG	150		**Gln**	CAG	235	
**Ile**	AUU	874		**Asn**	AAU	647	
**Ile**	AUC	379	trnI-GAU	**Asn**	AAC	274	trnN-GUU
**Ile**	AUA	562	trnI-CAU	**Lys**	AAA	865	trnK-UUU
**Met**	AUG	522	trn(f)M-CAU	**Lys**	AAG	367	
**Val**	GUU	473		**Asp**	GAU	619	
**Val**	GUC	182	trnV-GAC	**Asp**	GAC	209	trnD-GUC
**Val**	GUA	505	trnV-UAC	**Glu**	GAA	807	trnE-UUC
**Val**	GUG	196		**Glu**	GAG	372	
**Ser**	UCU	458		**Cys**	UGU	215	
**Ser**	UCC	328	trnS-GGA	**Cys**	UGC	106	trnC-GCA
**Ser**	UCA	296	trnS-UGA	**Stop**	UGA	17	
**Ser**	UCG	161		**Trp**	UGG	430	trnW-CCA
**Pro**	CCU	375		**Arg**	CGU	312	trnR-ACG
**Pro**	CCC	243		**Arg**	CGC	152	
**Pro**	CCA	291	trnP-UGG	**Arg**	CGA	311	
**Pro**	CCG	151		**Arg**	CGG	152	
**Thr**	ACU	507		**Arg**	AGA	436	trnR-UCU
**Thr**	ACC	236	trnT-GGU	**Arg**	AGG	221	
**Thr**	ACA	331	trnT-UGU	**Ser**	AGU	349	
**Thr**	ACG	173		**Ser**	AGC	176	trnS-GCU
**Ala**	GCU	593		**Gly**	GGU	539	
**Ala**	GCC	242		**Gly**	GGC	225	trnG-GCC
**Ala**	GCA	413	trnA-UGC	**Gly**	GGA	653	trnG-UCC
**Ala**	GCG	202		**Gly**	GGG	359	

*Numerals indicate the frequency of usage of each codon in 23430 in codons in 81 potential protein-coding genes.

### Comparison with Other Poaceae Chloroplast Genomes

**Figure 2:**
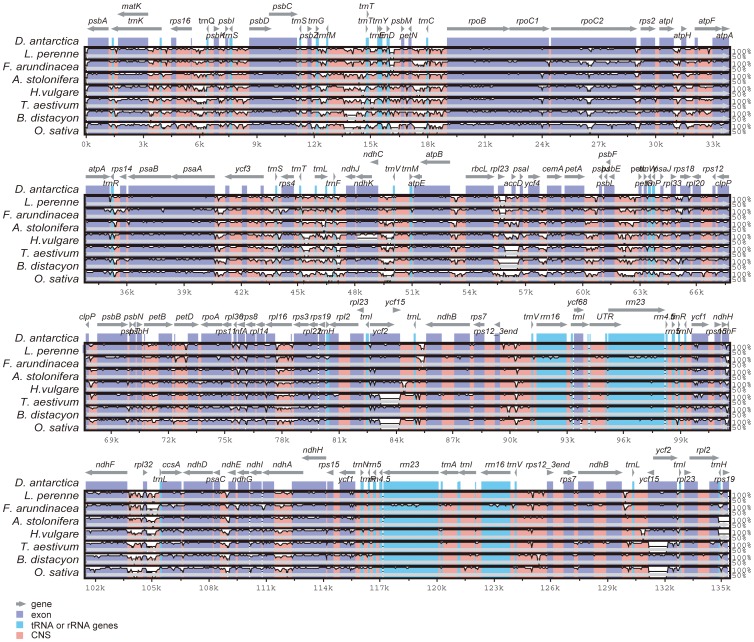
Sequence alignment of eight Poaceae chloroplast genomes. The top line shows genes in order (transcriptional direction indicated by arrows). The sequence similarity of the aligned regions between *Deschampsia antarctica* and the other seven species is shown as horizontal bars indicating the average percent identity between 50% and 100% (shown on the *y*-axis of the graph). The *x*-axis represents the coordinate in the chloroplast genome. Genome regions are color coded as protein-coding (exon), tRNA or rRNA, and conserved noncoding sequences (CNS).

The availability of multiple complete Poaceae chloroplast genomes provides an opportunity to compare sequence variation within the family at the genome-level. The sequence identity of seven Poaceae chloroplast genomes was plotted using the mVISTA program, with the annotation of *D. antarctica* as a reference (Figure 2, percent identity plot, as summarized in Table S3). The whole aligned sequences indicate that the Poaceae chloroplast genomes are rather conservative, although some divergent regions were found between these genomes. Similar to other plant species, the coding region is more conservative than the noncoding counterpart. Of all genes, *ycf1* appears to be the most divergent pseudogene. In addition, *rpl32*, *ycf2*, and *rpoC2* also displayed high sequence divergence. The noncoding regions showed a higher sequence divergence than the coding regions among the eight Poaceae chloroplast genomes. In the alignment sequences, several intergenic regions were found to display high divergence, including *trnG(UCC)*-*trnfM(CAU)*, *trnY(GUA)-trnD(GUC)*, *ndhF-rpl32*, and *rpl32*-*trnL(UAG)*. In addition, the intron sequences from *trnK(UUU)*, *trnL(UAA)*, and *ndhA* showed high sequence divergence.

**Figure 3:**
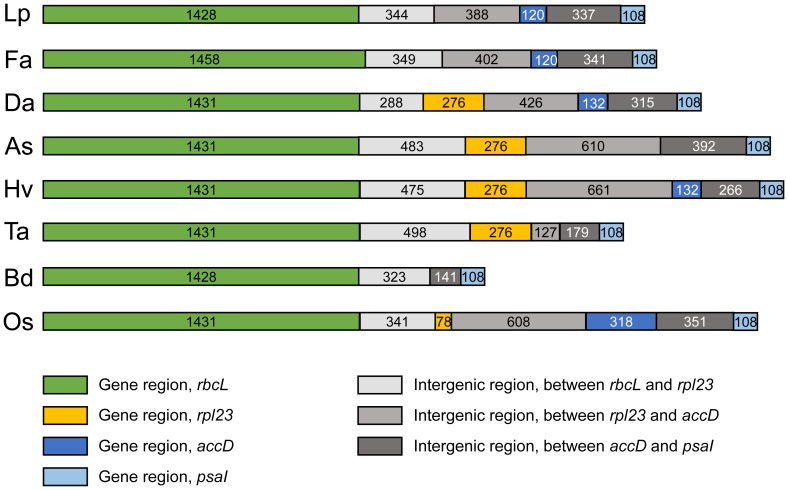
Comparison of the *rbcL-psaI* region among eight Poaceae species. The genes and intergenic regions between *rbcL* and *psaI* are indicated by boxes, with the length presented in bp. (Lp: *Lolium perenne*, Fa: *Festuca arundinacea*, As: *Agrostis stolonifera*, Hv: *Hordeum vulgare*, Ta: *Triticum aestivum*, Bd: *Brachypodium distachyon*, Os: *Oryza sativa* subsp. *japonica*).

The length variation was also examined among *D. antarctica* and the eight Poaceae chloroplast genomes. The most interesting region with length variation was the *rbcL*-*psaI* region, which contains four gene regions and three intergenic regions (Figure 3). The variation of gene region was detected in the presence of an *rpl23* translocation product and an *accD* pseudogene in the region between *rbcL* and *psaI*. The *rpl23* gene was absent from *L. perenne*, *F. arundinacea*, and *Brachypodium distachyon*, and was present in the five other analyzed Poaceae species, including *D. antarctica*. Remnants of the *accD* gene were detected in *D. antarctica*, *L. perenne*, *F. arundinacea*, and *Hordeum vulgare*. This pseudogene was identified in rice but was not predicted in the other species according to DOGMA. The variation in size of the intergenics regions was also detected among species of the Pooideae subfamily. Three intergenic regions occurred between the *rbcL* and *psaI* genes. The intergenic region between *rbcL* and *rpl23* ranged from 288 bp (*D. antarctica*) to 498 bp (*Triticum aestivum*). Between *rpl23* and *accD*, it ranged from 0 bp (*B. distachyon*) to 661 bp (*H. vulgare*), and between *accD* and *psaI*, it ranged from 141 bp (*B. distachyon*) to 392 bp (*Agrostis stolonifera*). In cases when a particular gene was absent, the boundaries of the intergenic regions were determined based on homologies between the species.

### Phylogenomic Analysis

**Figure 4:**
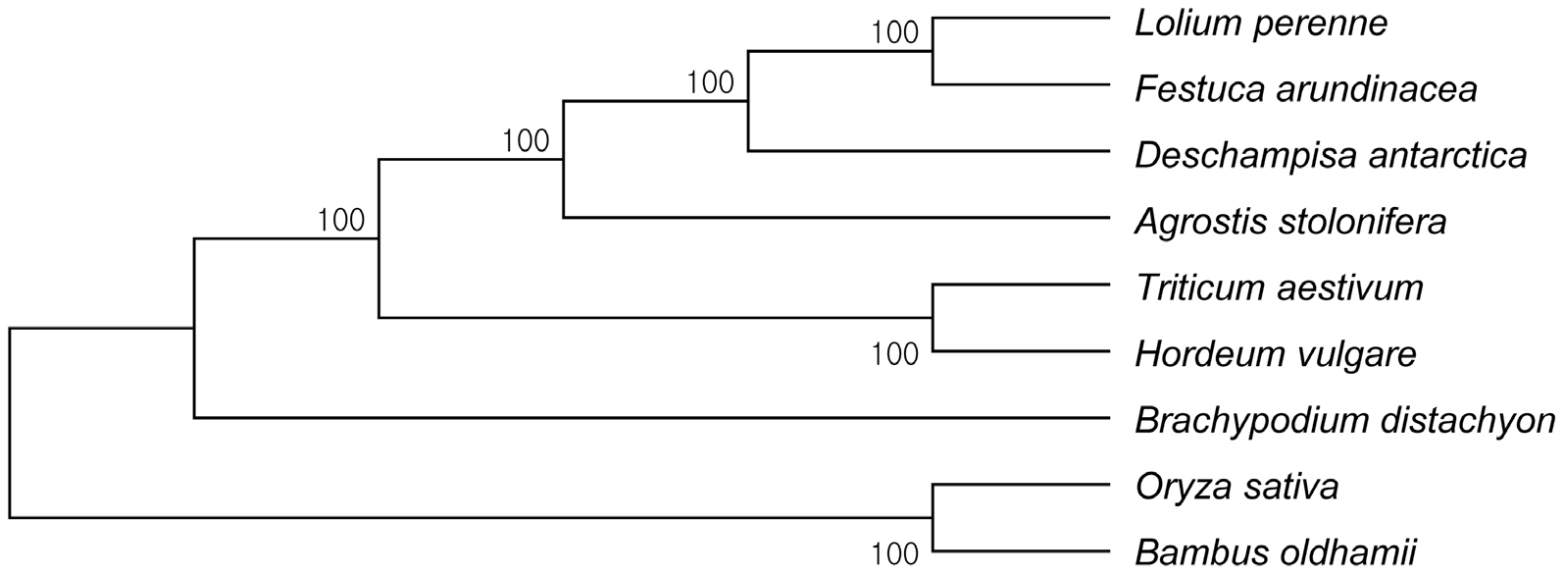
Maximum parsimony analysis of nine Poaceae species based on the whole plastome sequence. The plastome sequences of *Oryza sativa* and *Bambusa oldhamii* were included as outgroup species. The phylogenetic tree was drawn using MEGA5, and bootstrap support was achieved using 1,000 replicates.

Phylogenomic analysis of representatives from the Pooideae subfamily, including *D. antarctica*, produced a single, well-supported tree using maximum parsimony (Figure 4). The tree is well congruent with respect to species, and the two outgroup species belonging to the BEP clade (*Bambusa oldhamii* from Bambusoideae and *Oryza sativa* subsp. *japonica* from Ehrhartoideae) are basal to the remaining species in a separate resolved clade.

### Repeat Sequence Analysis

**Figure 5:**
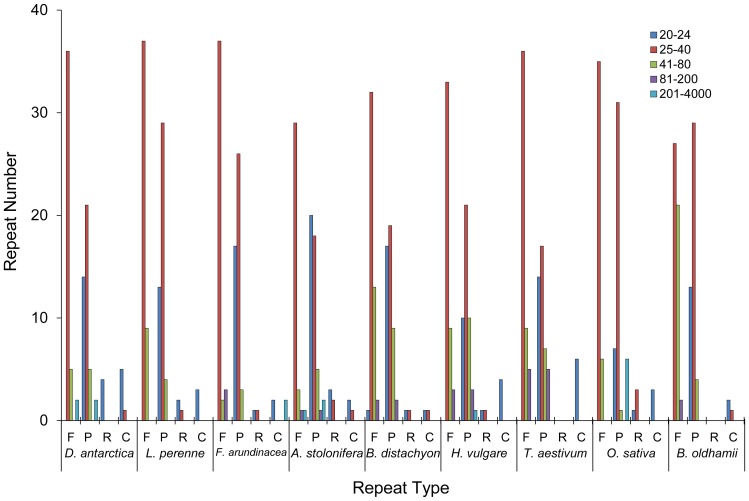
Repeat analysis in the *Deschampsia antarctica* chloroplast genome. Repeat sequences are compared among eight chloroplast genomes in the Poaceae family. To identify repeat sequences, the REPuter program was used. Repeats with length >20 bp and sequence identity *e*-value <10^−3^ were selected and categorized to four types based on their orientations (F: forward, P: palindromic, R: reverse).

Repeat regions of DNA are an important factor in genome recombination and rearrangement. We identified 69 repeats in *D. antarctica*, including 43 forward, 24 palindromic, and 2 reverse repeats with a length >20 bp and a sequence identity *e*-value <10^−3^, using the REPuter program (Table S4). Among the 69 repeats, 58 (84%) were 25–80 bp in length, 51 (63%) were 25–40 bp in length, and 10 (21%) were 41–80 bp in length. The repeats were mostly located in the intergenic sequences (54%), followed by coding sequences (37%) and intronic sequences (9%). The structure of the repeats in the other seven Poaceae species was also analyzed using REPuter. The majority of repeats in Poaceae species within the size range of 25–80 bp commonly are forward or palindromic (Figure 5). The total number of repeats varied among species (*D. antarctica*: 69, *L. perrene*: 72, *F. arundinacea*: 59, *A. stolonifera*: 50, *B. distachyon*: 60, *H. vulgare*: 67, *T. aestivum*: 79, *O. sativa*: 78, *B. oldhamii*: 74). The repeat pattern in *D. antarctica* was more similar with *L. perenne* and *F. arundinacea* in the Poeae tribe than with *B. oldhamii* from the Bambusoideae. For example, repeats in the size range of 41–80 bp represent ≤20% of the total number of repeats in species of the Pooideae subfamily, whereas they represent >28% of the total in *O. sativa* and *B. oldhamii*.

### Expression Analysis

We performed an expression analysis of the 81 chloroplast protein-coding genes using in-house RNA-seq data from leaf tissues of *D. antarctica* (Lee et al., unpublished data). The short reads were mapped to the *D. antarctica* chloroplast genome, and the numbers of reads corresponding to coding genes were calculated and normalized according to gene length (Table 4). The most abundant genes were *ndhC*, *psbJ*, *rps19*, *psaJ*, and *psbA*, with FPKM value >10,000. Thirteen genes (*ccsA*, *ndhI*, *rpoA*, *rpoC2*, *rps2*, *ndhA*, *ndhD*, *ycf1*, *rps11*, *rps3*, *ycf2*, *rpoC1*, and *rpoB*) had low expression, with FPKM value <100.

**Table 4:** RNA Expression of protein coding genes in the *Deschampsia antarctica* chloroplast genome.

locus ID	gene name	locus	FPKM	locus ID	gene name	locus	FPKM
DeanCp027	*ndhC*	48846–49209	87311	DeanCp032	*accD*	56279–56411	326
DeanCp037	*psbJ*	60890–61013	19529	DeanCp073	*rps15*	100781–101054	314
DeanCp064	*rps19*	79951–80233	13440	DeanCp076	*rpl32*	104531–104714	309
DeanCp043	*psaJ*	64095–64224	10915	DeanCp025	*ndhJ*	47535–48015	288
DeanCp002	*psbA*	83–1145	10274	DeanCp016	*atpI*	30197–30941	283
DeanCp042	*petG*	63234–63348	9557	DeanCp074	*ndhH*	101191–101431	254
DeanCp051	*psbN*	70154–70286	7916	DeanCp004	*rps16*	4488–5567	250
DeanCp011	*petN*	17020–17110	7370	DeanCp080	*ndhE*	109031–109337	202
DeanCp018	*atpF*	32063–33432	6499	DeanCp036	*petA*	59093–60056	188
DeanCp039	*psbF*	61282–61402	6371	DeanCp065	*rpl2*	80495–81980	185
DeanCp038	*psbL*	61143–61260	4812	DeanCp069	*ndhB*	85606–87851	165
DeanCp010	*psbM*	16638–16743	3750	DeanCp070	*rps7*	88150–88621	159
DeanCp006	*psbI*	7354–7465	3335	DeanCp060	*rpl14*	76697–77069	152
DeanCp050	*psbT*	69989–70106	3089	DeanCp023	*ycf3*	41199–43189	152
DeanCp040	*psbE*	61412–61664	3020	DeanCp068	*ycf15*	83879–84167	149
DeanCp052	*psbH*	70389–70611	2998	DeanCp045	*rps18*	65145–65658	144
DeanCp020	*rps14*	35628–35940	2224	DeanCp058	*infA*	75691–76042	144
DeanCp017	*atpH*	31349–31595	2138	DeanCp046	*rpl20*	65815–66175	144
DeanCp005	*psbK*	6761–6947	1970	DeanCp024	*rps4*	44159–44765	142
DeanCp009	*psbZ*	11675–11864	1948	DeanCp048	*clpP*	67131–67782	141
DeanCp030	*rbcL*	53858–55292	1782	DeanCp081	*ndhG*	109549–110080	136
DeanCp007	*psbD*	8635–9697	1333	DeanCp034	*ycf4*	57149–57707	134
DeanCp033	*psaI*	56726–56837	1308	DeanCp003	*matK*	1685–3221	133
DeanCp041	*petL*	62963–63059	1282	DeanCp061	*rpl16*	77186–78490	132
DeanCp049	*psbB*	68293–69820	1197	DeanCp059	*rps8*	76143–76554	128
DeanCp071	*ycf68*	93397–93832	1096	DeanCp063	*rpl22*	79429–79873	113
DeanCp079	*psaC*	108276–108522	1052	DeanCp035	*cemA*	58162–58861	110
DeanCp019	*atpA*	33523–35047	971	DeanCp075	*ndhF*	101464–103684	102
DeanCp054	*petD*	72341–73561	956	DeanCp077	*ccsA*	105547–106507	87
DeanCp057	*rpl36*	75472–75586	956	DeanCp082	*ndhI*	110198–110741	85
DeanCp044	*rpl33*	64666–64867	922	DeanCp055	*rpoA*	73770–74796	74
DeanCp001	*rps12*	66870–89475	840	DeanCp014	*rpoC2*	24536–28943	66
DeanCp021	*psaB*	36086–38291	676	DeanCp015	*rps2*	29236–29947	62
DeanCp022	*psaA*	38316–40569	558	DeanCp083	*ndhA*	110838–112939	58
DeanCp008	*psbC*	9644–11066	513	DeanCp078	*ndhD*	106654–108157	57
DeanCp084	*ndhH*	112940–114122	510	DeanCp072	*ycf1*	99622–100414	52
DeanCp053	*petB*	70745–72153	495	DeanCp056	*rps11*	74860–75292	48
DeanCp031	*rpl23*	55577–55853	419	DeanCp062	*rps3*	78636–79356	44
DeanCp047	*rps12*	125837–126080	402	DeanCp067	*ycf2*	82674–83874	39
DeanCp066	*rpl23*	81998–82280	400	DeanCp090	*ycf2*	131435–132638	39
DeanCp029	*atpB*	51509–53006	385	DeanCp013	*rpoC1*	22302–24333	31
DeanCp026	*ndhK*	48118–48856	342	DeanCp012	*rpoB*	19034–22265	29
DeanCp028	*atpE*	51099–51513	329				

A total of 247,904 reads mapped to the protein coding region. Among these, 89,675 (36.2%) and 73,054 reads (29.5%) were generated from genes encoding components of the cyclic electron transfer system and photosystem II (PSII) complex, respectively. In addition, among the 18 highly expressed genes (FPKM value >2,000), 10 genes were found to encode subunits of the PSII complex (*psbA*, *psbB*, *psbE*, *psbF*, *psbH*, *psbI*, *psbL*, *psbM*, *psbN*, and *psbT*). In contrast, *rpoA*, *rpoB*, *rpoC1*, and *rpoC2*, which encode plastid RNA polymerase, showed very low expression.

### RNA Editing

RNA editing is a sequence-specific posttranscriptional modification resulting in conversion, insertion, and deletion of nucleotides in a precursor RNA. Such modifications are observed across organisms. In plants, RNA editing has been reported to occur with C-to-U or U-to-C (rare) conversions in mitochondria and plastids [26].

In the *Deschampsia* chloroplast genome, we first predicted 37 RNA-editing sites out of 16 genes using the PREP-chloroplast program (Table S5). Using another method, we aligned read sequences from the RNA-seq data using variant searching tools comparing transcripts against a reference genome and confirmed 30 editing sites. The 30 nucleotide substitutions occur in 23 genes in the *D. antarctica* chloroplast genome, which results in 25 non-synonymous amino acid changes (Table 5). Of the substitutions, 17 (54.8%) were C-to-U conversions, resulting in 14 non-synonymous amino acid changes. In contrast, only 1 edit was a U-to-C conversion with synonymous base change. Although RNA editing of plant plastids has been shown to be conversions of C to U and U to C, we observed different versions of edits, including 3 A-to-Cs, 3 A-to-Gs, 3 G-to-As, 1 G- to- C, 1 U-to-A, 1 A-to-U, and 1 U-to-G in 13 sites.

**Table 5:** RNA editing sites in the *Deschampsia antarctica* chloroplast genome.

Gene	length	location from start	codonchange	amino acid change	Nucleotidechange	Number of reads*
*matK*	1536	1258	CAU>UAU	His>Tyr	C>U	U;11 (28.9%), C; 27 (71.1%)
*rpoB*	3231	398	CGC>CAC	Arg>His	G>A	A:4 (25%), G:10 (62.5%), U:1(6.3%),
*rpoC1*	2031	603	GAA-GAU	Glu>Asp	A>U	A:19 (74.1%), U:7 (25.9%)
*rpoC1*	2031	612	GCG-GCA	Ala->Ala	G>A	A:7 (24.1%), G:22 (75.9%)
*rpoC2*	4407	650	AUA>AGA	Ile>Arg	U>G	G:2 (20.0%. U:8 (80%)
*atpA*	1524	334	UUG>CUG	Leu>Leu	U>C	C:13 (43.3%), U:17(56.7%)
*atpA*	1524	367	AUA>GUA	Ile>Val	A>G	A:23 (76.7%), G:7(23.3%)
*atpA*	1524	933	GAA>GAC	Glu>Asp	A>C	A:41(54.7%), C:34(45.3%)
*atpA*	1524	1148	UCA>UUA	Ser>Leu	C>U	C:2(2.9%), U:66 (97.1%)
*ycf3*	513	44	UCC>UUC	Ser>Phe	C>U	U:19 (100%)
*rps4*	606	588	UAU>UAA	Tyr>stop	U>A	A:55 (66.3%),U:28 (33.7%)
*rps4*	606	580	GUG>CUG	Val>Leu	G>C	G:31(36%), C:55 (64%)
*rps4*	606	370	AAU>GAU	Asn>Asp	A>G	G:3 (42.9%), A:4 (57.1%)
*ndhJ*3′ UTR			AAU>AAC	Asn>Asn	A>C	A:4 (30.8%), C: 9 (69.2%)
*ndhJ*	480	480	UGA>UGG	stop>Trp	A>G	A:4 (30.8%), G: 9 (64.3%)
*ndhK*	738	125	CCA>CUA	Pro>Leu	C>U	C:2(9.5%), U:19 (90.5%)
*ndhC*	363	13	CAC>UAC	His>Tyr	C>U	C:3(50%). U:3 (50%)
*psbL*	117	111	UUC>UUU	Phe>Phe	C>U	C:2 (15.4%), U: 10 (76.9%), G: 1(7.7%)
*petL*	96	56	CCA>CUA	Pro>Leu	C>U	U:2 (100%)
*rpl20*	360	308	UCA>UUA	Ser>Leu	C>U	C:5 (45.5%), U:6 (54.5%)
*psbB*	5127	867	AGC>AGU	Ser>Ser	C>U	C:25 (83.3%), U:5 (16.7%)
*petB*	648	611	CCA>CUA	Pro>Leu	C>U	U:19 (100%)
*rpoA*	1026	527	UCC>UUC	Ser>Phe	C>U	C:2 (18.2%), U:9 (81.8%)
*rps8*	411	182	UCA>UUA	Ser>Leu	C>U	C:1 (7.7%), U:12 (92.3%)
*rpl16*	411	250	GGC>ACG	Gly>Ser	G>A	G:2 (33.3%), A:4 (66.7%)
*rps3*	720	30	UUC>UUU	Phe>Phe	C>U	C:6 (30%), U:14 (70%)
*ndhD*	1503	878	UCA>UUA	Ser>Leu	C>U	C:1 (9.1%), U:10 (90.9%)
*ndhG*	531	347	CCA>CUA	Pro>Leu	C>U	U: 29 (100%)
*ndhA*	1089	722	GCA>GUA	Ala>Val	C>U	C: 9 (81.8%), U: 2 (18.2%)
*ndhA*	1089	474	UCA>UUA	Ser>Leu	C>U	C: 2 (10.5%), U: 17 (89.5%)

*indicates the number of reads with an alternate base and the number of reads with the same base as the reference.

We calculated the ratio between the number of reads with an alternate base and the number of reads with the same base as the reference. The percentages of the conversion rates of each edit varied with the locus (16–100%) (Table 5). However, some edits with C-to-U conversion in several genes showed very high editing rates (>90%), especially for *atpA*, *ycf3*, *ndhK*, *petB*, *rpoA*, *rps8*, *ndhD*, *ndhG*, and *ndhA*, suggesting that the edited RNAs for these gene are common forms in the processed RNA pools in *D. antarctica*.

### Discovery of Plastid Small Noncoding RNA in *D. antarctica*

**Figure 6:**
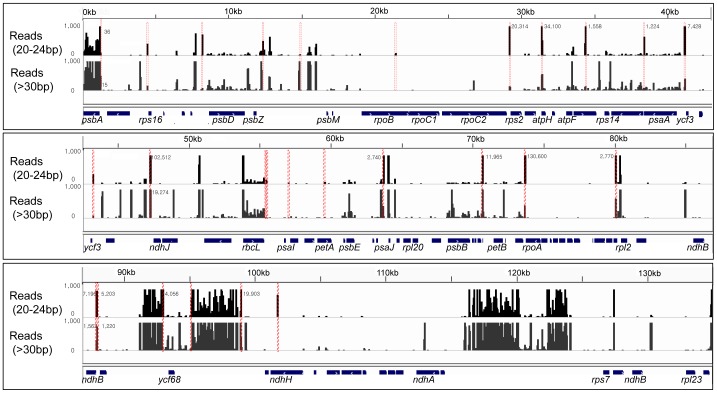
Distribution of plastid small RNAs in the *Deschampsia antarctica* chloroplast genome. The reads from small RNA-seq were divided into two groups according to the length (20–24 nt and >30 nt) and aligned to the *D. antarctica* chloroplast genome with 100% identity. The distributions of reads were compared between the two groups. In total, 12,753,636 reads were distributed unevenly in the chloroplast genome with high density in the coding regions of *psbA* and *rbcL*, intergenic regions, and inverted repeat regions in which most of the rRNA genes exist. The 27 loci enriched with 20–24 nt RNAs are indicated in red, along with the number of reads. The *y*-axis shows the number of reads (from 0 to 1000).

Numerous small noncoding RNAs have been identified in the nuclear genomes of bacteria and eukaryotes. Small noncoding RNAs are also transcribed from mitochondria and plastid genomes [27]–[29]. In this study, we screened for small noncoding RNAs from our deep sequencing data in the small RNA library generated from *D. antarctica* leaf tissues. The reads between 20 and 24 nt in length were mapped to the chloroplast genome with 100% identity. In total, 12,753,636 reads were distributed unevenly throughout the chloroplast genome (Figure 6), including coding regions of *psbA* and *rbcL*, intergenic regions, regions encoding several tRNA genes, and inverted repeat regions in which most of the rRNA genes exist. To exclude RNA fragments that may have been generated from abundant RNA species, we compared the distribution of reads that were 20–24 nt in length with those longer than 30 nt. As a result, we identified 27 loci where short noncoding RNAs (sRNAs) of 20–24 nt length with unique sequences were abundantly expressed (Table 6).

**Table 6:** Distribution of small RNAs in the chloroplast genome of *Deschampsia antarctica*.

Loca-tion	start	end	F/R*	core sequence	length			reads number	At	Os	Hv
*psbA*5′ end	1229	1209	R	AACAAGCCTTCTATTATCTA		20	36		+		
*trnK*-*rps16*	4378	4356	R	TGTCGTGCCAATCCAACATAAGCC		23	819			+	+
*psbI*-*psbD*	8129	8150	F	TTCCTTAGACTTAGACCGCGC		21	1200				
*trnG*-*trnfM*	12314	12333	F	ACCGTATCCCTTACTATTCT		20	1056				
*trnT*3′ end	14786	14806	F	GGTTCAAATCCGATAAAGGGC		21	148				
*rpoB* CDS	21313	21332	F	CGTCGTATATCGCGGAAGCT		20	166				
*rpoC2*-*rps2*	29130	29147	F	ATTTCAAGCTATTTCGGA		18	20314				+
*atpH*5′ end	31305	31325	F	ATTGTATCCTTAACCATTTCT		21	34100		+	+	+
*atpA* CDS	34274	34297	F	TTATGTACCGCGAACGGCATA		21	1558				
*psaB*5′ end	38291	38315	R	AGGAGGATTTGAAAGGCATTA		21	1224				
*ycf3*3′ end	41108	41088	R	TTCATTATATCGCTTTCTTCT		21	7428				+
*ycf3*5′ end	43251	43232	R	TTTGTTTTTATGTTATTTTG		20	450		+		+
*trnF*-*ndhJ*	47285	47265	R	CTTTGTATCGCGCGCATGACT		21	102512				
*rbcL*3′ end_1	55406	55426	F	CTCGGCTCAATCTTTTTTAGA		21	111		+	+	
*rbcL*3′ end_2	55424	55431	F	AAAAAAAAGATTGAGCCGAAT		21	160		+	+	
*psaI*-*ycf4*	57004	57024	F	TGAATAGAAAGTCAATGTATC		21	120				
*petA* CDS	59574	59553	R	TTTCACTATATTTCTTACCGGG		22	230				
*trnP*5′ end	63758	63739	R	AGGGATGTAGCGCAGCTTGG		20	2740				
*psbH*-*petB*	70702	70721	F	GGTAGTTCGACCGCGGAATT		20	11965		+	+	+
*petD*-*rpoA*	73658	73677	F	TTATTATGATCCATTTCGCG		20	130600			+	+
*rps19* CDS	80081	80098	F	ATGAATCGCGATTGTATG		18	2770				
*ndhB*5′ end 1	87859 (127454)	87839 (127474)	R	ACTAATTCATGATCTGGCATG		21	7196		+	+	+
*ndhB*5′ end 2	87863 (127450)	87843 (127470)	R	AGTTACTAATTCATGATCTGG		21	5203		+	+	+
*rrn16*3′ end	92921 (122393)	92941 (122373)	F	GGTGCGGCTGGATCACCTCCT		21	4056				
*trnA* intron	95093 (120221)	95113(120201)	F	CTTAGCGGATACTATGATAGC		21	982				
*trnR*3′ end	98927 (116387)	98946 (116368)	F	GTGTCGGGGGTTCGAATCCC		20	19903			+	
*ndhF* CDS	101690	101709	F	ATAACCGCGATTATATGACC		20	1149				

*F/R: Direction of transcripts (F: forward, R: reverse).

**Figure 7:**
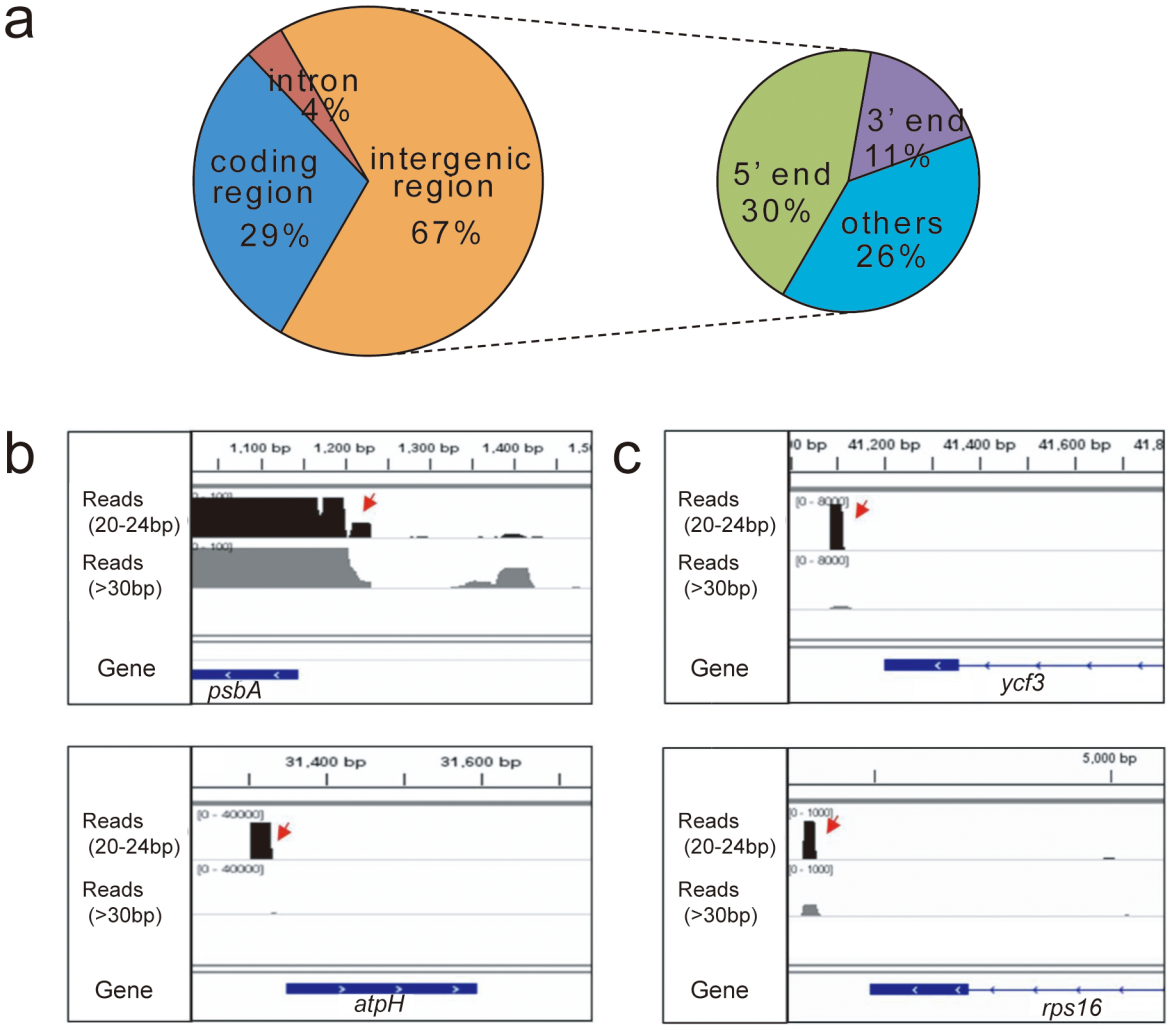
Relative locations of small RNAs in the *Deschampsia antarctica* chloroplast genome. **a** Relative locations of plastid small RNAs according to the gene structure; **b** examples of small RNAs located proximal to the 5′ ends of the coding genes; **c** examples of small RNAs located proximal to the 3′ end of the coding genes.

The *D. antarctica* plastid sRNAs were not evenly distributed throughout the genome. The relative positions of the sRNAs showed that 19 of 27 (71%) were located in the noncoding regions (18 in intergenic regions and 1 in an intronic region). In particular, 30% and 11%, respectively, of the intergenic sRNAs were located at the 5′- and 3′-ends of genes (>100 bp from the start or termination codons) (Figure 7). Fifteen (55.6%) sRNAs were located within −150 to +50 bp from the start codon of genes, suggesting that proximity to the 5′-ends of genes is important.

**Figure 8:**
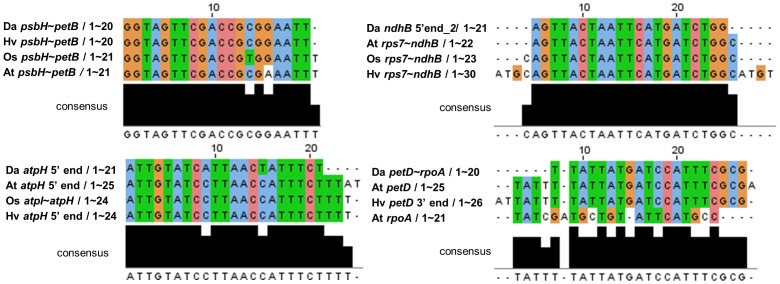
Sequence conservation among orthologs of plastid small RNAs. To determine if the identified sRNAs are evolutionarily conserved, *Deschampsia antarctica* sRNAs were compared with the plastid sRNAs identified in *Arabidopsis*, rice, or barley [28], [29]. The sequence aligments of sRNAs which have >90% sequence homology are shown. The multiple sequence alignments were performed with ClustalW2 algorithm (http://www.ebi.ac.uk/Tools/msa/clustalw2/) and visualized with Jalview program [41]. The consensus sequences between ortholog sRNAs were shown at the bottom of each alignment.

To determine if the identified sRNAs are evolutionarily conserved, we compared the sequences of 27 sRNAs in *D. antarctica* with the sRNAs reported for other plant species by multiple sequence alignment [28], [29]. In total, we found that 13 sRNAs have orthology with the plastid sRNAs found in *Arabidopsis*, rice, or barley (Figures 8, Figure S1, and Table 6). Among the pairs identified, four sRNAs (*psbH*-*petB*, *atpH* 5′end, *ndhB* 5′end, and *petD_rpoA*) showed >90% sequence homology, and their locations within the genome were the same in all of the species examined, suggesting these plastid sRNAs may be evolutionarily conserved across angiosperms (Figure 8).

## Discussion

We obtained the completed sequence of the chloroplast genome of *D. antarctica* using whole genome sequencing data from total genomic DNA from leaves. As previous studies have reported, aligning all the reads against the plastid genome database allow the rapid and efficient assembly of the chloroplast genome [8], [30], [31]. By this method, we identified 1.2% of the total genomic reads as chloroplast-related sequences.

The chloroplast genome of *D. antarctica* has the typical features found in the genomes of other Poaceae species. The size of its genome and the ratio of GC content is 135,362 bp and 38.3%, respectively, similar to other Poaceae species. The subfamily Pooideae, which includes one-third of all grass species, has been divided into 13 tribes [14], but recent analyses have demonstrated wide variations between them. For example, neither Poeae nor Aveneae are monophyletic, and the components of these two groups are intermixed within a clade [13], [32]. Traditional morphological phylogenetic studies placed *Deschampsia* within the tribe Aveneae. However, molecular studies inferred alternative phylogenetic positions of *Deschampsia* (i.e., Aveneae or Poeae), depending on the target sequences used for examination or the parameters used for grouping [12], [13], [32]–[35]. In this study, we revised the phylogenetic position of *D. antarctica* using complete sequences of chloroplast DNA. A comparative analysis based on both whole plastome and open reading frame sequences of coding genes suggest that *D. antarctica* is more closely related with species in the Poeae tribe than the Aveneae tribe. This is in agreement with the results of Davis and Soreng [13], Catalan et al. [33], and Nadot et al. [34], in which *Deschampsia* forms a closer relationship with species of the Poeae than with those of Aveneae, as suggested by Souto et al. [12] and Hsiao et al. [35]. However, in our genome structure analysis, we found an interesting region (*rbcL*–*psaI*) where both the *rpl23* translocation product and *accD* pseudogene were found. This appears to be specific to *Deschampsia*, since other Poeae or Aveneae species have kept only one remnant of *accD* or *rpl23* in the region, suggesting that this region could be molecular evidence for an intermixed lineage of *Deschampsia.*

For the transcriptome analysis of the chloroplast genome, we utilized RNA-seq data from libraries generated by two preparation methods (mRNA-seq and small RNA-seq). We found that a significant proportion of the reads from RNA-seq data represent the organelle derived sequences, suggesting that the eukaryotic RNA-seq results are very good resources for a functional study of genes in organelles.

The transcriptome analysis of *D. antarctica* plastid RNAs revealed several interesting aspects of RNA metabolism. First, a search of the variant transcripts revealed numerous RNA-editing sites in the *D. antarctica* chloroplast genome. RNA editing has been observed in the chloroplasts of extant descendants of early land plants other than liverworts and mosses. In angiosperm plastids, RNA editing is mostly restricted to a C-to-U conversion, and the conversion occurs at about 30 different positions, whereas hornworts and fern plastids extensively edit U-to-C as well as C-to-U at >300 different positions [36]. A comparative analysis of eight land plants, including hornworts, ferns, and seed plants, suggested that chloroplast RNA editing is of monophyletic origin and evolved as a system to generate new variations [37]. Our transcriptome analysis revealed *in situ* editing sites beyond those predicted by computational tools (Table 4 vs. Table S5). According to the variant transcript search, the major form of RNA-editing is C-to-U conversion (54.8%), and the conversion rate of C-to-U edits (>90%) is much higher than those of other edits. Some edits with C-to-U conversion in several genes, such as *atpA*, *ycf3*, *ndhK*, *petB*, *rpoA*, *rps8*, *ndhD*, *ndhG*, and *ndhA*, have been reported in other species [37], indicating that these edits are functionally conserved in plants. Comparison between the whole genome DNA and transcriptome data also showed that various versions of edits exist and that their respective conversion rates differ. The difference in conversion rates among edits might be the result of tissue-specific, gene-specific, or developmental stage-specific RNA-editing patterns. Considering that mitochondrial RNA editing occurs with developmental and tissue specificity in plants [38]–[40], exploring whether tissue-disparity exists in plastid RNA-editing and the regulatory mechanisms that underlie it would be worthwhile.

We identified 27 plastid small noncoding RNAs in the *D. antarctica* chloroplast genome by high-resolution mapping of the transcriptome data. In *Arabidopsis*, rice, maize, and barley, small RNAs are expressed in plastids and their sequences correlate with the termini of processed mRNA [28], [29]. These studies also suggested that the small RNAs are footprints of the RNA-binding pentatricopeptide repeat (PPR) proteins, which protect RNAs from exonucleolytic degradation. Our results support this hypothesis. We observed a large amount of small RNAs expressed in the *D. antarctica* plastid, and these RNAs were not randomly distributed but were located in intergenic regions preferentially near the 5′- or 3′-ends of coding regions. This suggests that many small RNAs are evolutionarily conserved in their sequences and locations, which might have resulted from the functionally conserved gene regulatory system of higher plants.

## Conclusions

Using Illumina high-throughput sequencing technology, we obtained the complete sequence of the *D. antarctica* chloroplast genome. This is the first chloroplast genome sequenced from a plant species endemic to Antarctica. Sequence divergence analysis with other plastomes of the BEP clade in the grass family suggests a sister relationship between *D. antarctica* and two species of the Poeae tribe, *F. anrundinacea* and *L. perenne*. In addition, we conducted high-resolution mapping of the chloroplast-derived transcripts resulting from RNA-seq data. As a result, we could make an expression profile for 81 protein-coding genes and proposed *ndhC*, *psbJ*, *rps19*, *psaJ*, and *psbA* as the most highly expressed chloroplast genes in *D. antarctica*. Analysis of small RNA-seq revealed that 27 small noncoding RNAs are preferentially located close to the 5′- or 3′-ends of genes. Also, >30 RNA-editing sites were found in the *D. antarctica* chloroplast genome, with a predominance of C-to-U conversions. These will be very useful for molecular phylogeny studies of the evolution of Antarctic plants and for transcriptome studies specific to plant organelles.

## Supporting Information

Figure S1 **Comparison of small RNA sequences from different species.**
(TIF)

Table S1 **List of primer pairs used in sequence verification and improvement of the** ***Deschampsia antarctica*** **chloroplast genome.**
(XLSX)

Table S2 **The GenBank accession numbers of all eight chloroplast genomes used for phylogenetic analysis.**
(XLSX)

Table S3 **Comparison of homologs between the** ***Deschampsia antarctica*** **chloroplast genome and** ***Lolium perenne*** **(Lp),** ***Festuca arundinacea*** **(Fa),** ***Agrostis stolonifera*** **(As),** ***Hordeum vulgare*** **(Hv),** ***Triticum aestivum*** **(Ta),** ***Brachypodium distachyon*** **(Bd), and** ***Oryza sativa*** **subsp.** ***japonica*** **(Os) by the percent identity of coding and noncoding regions.**
(XLSX)

Table S4 **Repeat sequences in the** ***Deschampsia antarctica*** **chloroplast genome.**
(XLSX)

Table S5 **The 37 RNA-editing sites predicted by the PREP-cp program.**
(XLSX)
