# Correction: Combined Analysis of the Chloroplast Genome and Transcriptome of the Antarctic Vascular Plant *Deschampsia antarctica* Desv

**DOI:** 10.1371/journal.pone.0101100

**Published:** 2014-06-17

**Authors:** The PLOS ONE Staff

There are errors in the Funding section. The correct funding information is as follows: This work was supported by the Korea Polar Research Institute (Grant PE14070). The funders had no role in study design, data collection and analysis, decision to publish, or preparation of the manuscript.
