# Supplementary figures and images for: Combined Analysis of the Chloroplast Genome and Transcriptome of the Antarctic Vascular Plant Deschampsia antarctica Desv
Source: PLoS One. 2014 Mar 19;9(3):e92501. doi: 10.1371/journal.pone.0092501 (PMC3960257; doi:10.1371/journal.pone.0092501)

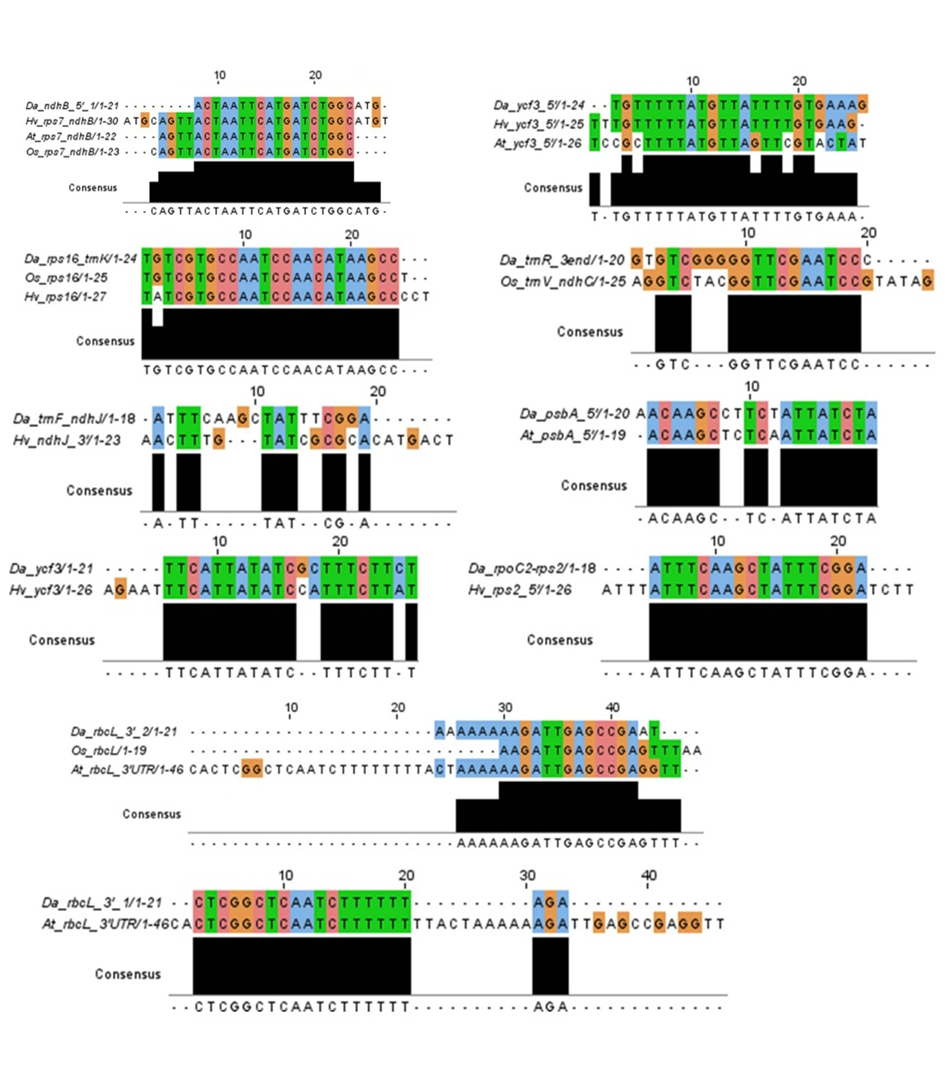

Supplement: Figure S1 — Comparison of small RNA sequences from different species. (TIF) [file pone.0092501.s001.tif]
